# Flight muscles degenerate by programmed cell death after migration in the wheat aphid, *Sitobion avenae*

**DOI:** 10.1186/s13104-019-4708-z

**Published:** 2019-10-21

**Authors:** Honglin Feng, Xiao Guo, Hongyan Sun, Shuai Zhang, Jinghui Xi, Jiao Yin, Yazhong Cao, Kebin Li

**Affiliations:** 1 State Key Laboratory for Biology of Plant Diseases and Insect Pests, Institute of Plant Protection, Chinese Academy of Agricultural Science, NO. 2 Yuanmingyuan Xilu, Haidian District, Beijing, 100193 China; 2 College of Plant Science, Jilin University, No. 5333 Xi’an Road, Changchun, 130062 Jilin China; 3 Chongqing Academy of Agricultural Sciences, Baishiyi, Jiulongpo District, Chongqing, 401329 China; 4 Present Address: Boyce Thompson Institute, 533 Tower Road, Ithaca, NY 14853 USA

**Keywords:** Aphid, *Sitobion avenae*, Flight muscle, Ubiquitin-ribosomal S27a, Programmed cell death

## Abstract

**Objective:**

Previous studies showed that flight muscles degenerate after migration in some aphid species; however, the underlying molecular mechanism remains virtually unknown. In this study, using the wheat aphid, *Sitobion avenae*, we aim to investigate aphid flight muscle degeneration and the underlying molecular mechanism.

**Results:**

*Sitobion avenae* started to differentiate winged or wingless morphs at the second instar, the winged aphids were fully determined at the third instar, and their wings were fully developed at the fourth instar. After migration, the aphid flight muscles degenerated via programmed cell death, which is evidenced by a Terminal deoxynucleotidyl transferase dUTP-biotin nick-end labeling assay. Then, we identified a list of differentially expressed genes before and after tethered flights using differential-display reverse transcription-PCR. One of the differentially expressed genes, ubiquitin-ribosomal S27a, was confirmed using qPCR. Ubiquitin-ribosomal S27a is drastically up regulated following the aphids’ migration and before the flight muscle degeneration. Our data suggested that aphid flight muscles degenerate after migration. During flight muscle degeneration, endogenous proteins may be degraded to reallocate energy for reproduction.

## Introduction

Many aphids develop wing polyphenism, and winged aphids explore new habitats by migration [1]. After winged aphids migrate to new host plants and/or the onset of larviposition, the indirect flight muscles (IFM) degenerate [2–6].

IFM degeneration has been previously depicted as regulated processes in insects including some aphids [7, 8]. The regulation of IFM degeneration involves multiple factors: juvenile hormone (JH); neural factors; and specific proteins. (i) JH treatment induces IFM degeneration [8–10]. (ii) Denervated muscles in the presence of JH initiated degeneration earlier than the innervated muscles in cricket [11]. (iii) specific proteins are induced despite the overall decreases of protein synthesis. Ubiquitin, a marker for programmed cell death (PCD), accumulates when aphid IFM undergoes degeneration [7, 8]. Although IFM degeneration has been suggested as an active PCD, the underlying molecular mechanism remains unclear.

The wheat aphid, *Sitobion avenae*, migrates from southern to northern China and causes ~ 10% wheat yield losses every year [12]. Here, we investigated *S. avenae* wing development and IFM degeneration, and identified some differentially expressed genes pre-/post-migration. We further analyzed the dynamic expression of ubiquitin-ribosomal S27a (*RPS27a*) during IFM degeneration.

## Main text

### Methods

#### Aphids

We generated an isogenic *S. avenae* population using a single wingless aphid. Aphids were raised on wheat seedlings at 22 °C with a 16 h/8 h light/dark photoperiod. Winged aphids were obtained by manipulating aphid densities. Under low-density, one wingless aphid was reared on a joint-stage wheat to maintain the wingless morph. Under high-density, 80 wingless adult aphids were reared on one ripe wheat to induce the winged morph [6].

To investigate IFM degeneration, we collected the winged aphids every 24 h from eclosion (0 day), to migration (5th day), to reproduction (8th day), until death was observed. For each timepoint, half of the aphids were collected for morphological, histological, and apoptosis examinations. The other half were dissected for qPCR following a freeze-drying procedure [13].

#### Morphological examination

We examined the external morphology of aphid thorax using scanning electron microscope (SEM). Aphids were fixed in 3% glutaraldehyde for 24 h and transferred to 1% osmic acid. Then aphids were saturated with ethanol, exchanged using isopentyl acetate, and dried in a Hitachi CO_2_ Critical Point Dryer. Aphids were then coated with gold in a sputter coater (Hitachi, IB-5) and imaged under a Hitachi S-570 SEM (Additional file 1: Figure S1).

We examined the internal morphology of aphid thorax using histological staining. Aphids were fixed in 4% paraformaldehyde for 4 h. The specimens were dehydrated in a serial of ethanol solutions (70%, 80%, 90%, 100%, 10 min/each), cleared in xylene, and embedded in paraffin. Serial sections were cut and stained with hematoxylin and eosin for imaging using a Carl Zeiss Primo Star Microscope (Additional file 1: Figure S1).

#### Terminal deoxynucleotidyl transferase dUTP-biotin nick-end labeling (TUNEL) assay

To examine apoptosis, we performed a TUNEL assay using an in situ apoptosis detection kit (Boster, China). Briefly, paraffin-embedded aphids were sliced into 5 μm sections, which were rehydrated in xylene for 20 min and a serial of ethanol solutions (100%, 90%, 80%, 70%, 10 min/each). Then all specimens were permeabilized using Proteinase K (1:200) for 10 min and quenched using 3% H_2_O_2_ for 10 min. Quenched specimen were labeled with TdT Labeling Reaction Mix (TdT:DIG-dUTP:Buffer = 1:1:18) for 2 h at 25 °C. After wash with 0.01 M TBS buffer, specimens were incubated with anti-DIG antibody (1:100) for 30 min. Specimens were then incubated with SABC (1:100) for 30 min and with Diaminobenzidine for 15 min, then counterstained with Hematoxylin for 3 min. Finally, specimens were washed with 100% ethanol, 100% xylene, and mounted for imaging.

Previous study suggested that feeding induces JH secretion, which triggers IFM degeneration [5]. Therefore, we examined the IFM degeneration on fasted aphid as a control. For fasting, winged aphids were transferred to water-soaked sponges. Every 6 h, aphids were transferred to rearing plants for 2 h to avoid death.

#### Differential-display reverse transcription-PCR (DDRT-PCR)

To identify differentially expressed genes pre-/post-migration, we performed tethered flight using 3–4 days post-eclosion aphids. For pre-migration, aphids were tethered (not flighted) and flash-frozen. For post-migration, aphids were tethered and flighted for 24 h using a flight-mill program [14]; flighted aphids were flash-frozen.

Total RNA was extracted from single aphid using Easy-Spin Total RNA Rapid Extraction Kit (Biomad) and reverse-transcribed by PrimeScript™ 1st-Strand cDNA Synthesis Kit (Takara). For DDRT-PCR, we designed three one-base anchored oligo-dT 3′ primers and eight arbitrary 5′ primers (13-mers) according to the GenHunter RNAimage DD Kit (3 × 8 pairs) (Additional file 2: Table S1). Each PCR reaction was composed of 10 μl 2× PCR mix, 1 μl 5′ primer, 1 μl 3′ primer, 2 μl cDNA, and 6 μl ddH_2_O. The PCR program was 94 °C for 1 min, followed by 30 cycles of 94 °C for 30 s, 48 °C for 1 min and 72 °C for 1 min, and a final extension step at 72 °C for 5 min. Then 2 μl PCR products of each reaction were used as template for a second round of PCR. The final PCR products were visualized using 6% SDS polyacrylamide gel electrophoresis (Additional file 3: Figure S2). Differentially expressed genes were excised for sequencing, obtained sequences were annotated on NCBI.

#### RPS27a dynamic expression

The sequence of *S. avenae RPS27a* was obtained using a rapid amplification of cDNA ends method (3′-RACE). Partial *S. avenae RPS27a* sequence containing the start codon was amplified using Ub-F/Ub-R primers designed based on *A. pisum RPS27a*. The complete C-terminal of *RPS27a* was obtained using a RACE kit (Takara) with gene specific primers and the kit-provided outer/inner primers (Additional file 4: Table S2). To check the conservation of *RPS27a*, the nucleotide and deduced amino acid sequences were aligned with homologous from other insects using DNAMAN (Additional file 5: Figure S3).

We quantified the *RPS27a* expression during IFM degeneration using qPCR in tissues including head, thorax, and abdomen. Briefly total RNA was extracted from tissues pooled from ~ 10 aphids, then same amount of RNA (0.1 μg) for each sample was used for cDNA Synthesis. The qPCR reaction was 25 μl containing 12.5 μl 2*PCR SuperMix, 0.5 μl Passive Reference DYE, 0.5 μl of each qPCR primer (Additional file 4: Table S2), 1 μl cDNA and ddH_2_O. The qPCR program was 2 min at 95 °C followed by 45 cycles of 15 s at 95 °C, 15 s at 55.5 °C and 30 s at 72 °C, and then melt curve under 55–95 °C 0.5 °C + (80 cycles). To calculate gene expression, a credible standard curve was constructed using a series of 10× dilutions of a standard sample. Each experiment included 3–4 technical replicates for each sample and repeated three times.

### Results

#### Flight muscle development in *S. avenae*

The external and internal structures were similar in the winged and wingless aphids at the 1^st^ instar; wing primordia were observed in the internal structures of both morphs (Additional file 1: Figure S1A, a). From the 2nd instar, wing primordia developed and enlarged in the winged morph, but disappeared in the wingless morph (Additional file 1: Figure S1b, b′). At the 3rd instar of wing aphids, swollen structures appeared and later developed into wing bud (Additional file 1: Figure S1C, C′). IFM fibers also differentiated; the corresponding area was occupied by fat bodies in the wingless morph hereafter (Additional file 1: Figure S1c, c′). To the 4th instar, wing buds enlarged into a plate shape and the wing epithelia were folded in a complicated structure, differentiating the forewings and hindwings (Additional file 1: Figure S1d′). To adults, the wings were fully developed; wing hair sensilla were also seen in the winged morph (Additional file 1: Figure S1E, F).

#### IFM degeneration after *S. avenae* migration

**Fig. 1:**
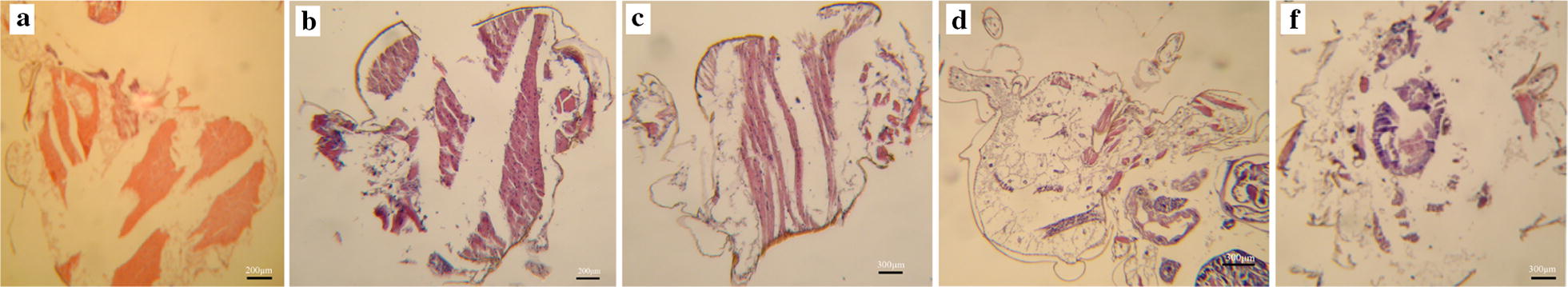
Breakdown of flight muscles of alatae aphids. Mesothoraces of alatae aphids at various stages were fixed, sectioned at 5 μm thickness and stained on the **a** 1st day, **b** 5th day, **c** 9th day, **d** 11th day, and **e** 13th day after the final ecdysis

The IFM of an alatae adult was plump at the 1st day after eclosion till the 5th day (Fig. 1a, b). From the 9th day, the IFM started to degenerate. The myofibrils appeared to be thin. The diameter of the myofibrils was reduced, and the volume of the interfibrillar sarcoplasmic region increased (Fig. 1c); the degradation continued through the 11th day (Fig. 1d). To the 13th day, the IFM was degenerated completely (Fig. 1e). The contractile fiber vanished, and the intact IFM was not visible.

#### IFM degeneration after migration is PCD

**Fig. 2:**
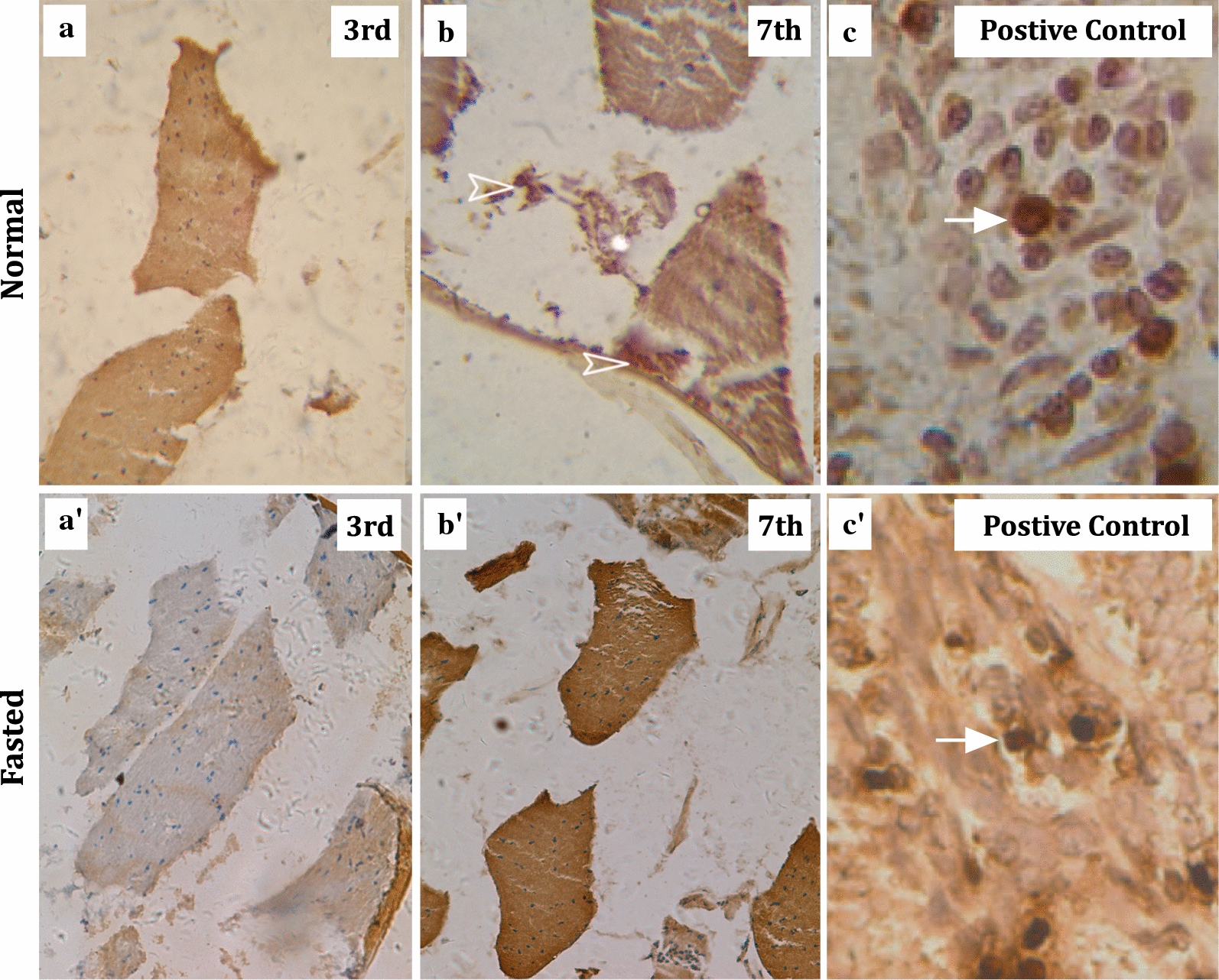
Cross-sections of the mesothoracic flight muscle at various stages. The apoptotic wing degeneration occurs at 7th day after the final ecdysis. All the mesothoraces were sectioned at 5 μm thickness. Images for the TUNEL assays are shown for the 3rd day (**a**, **a′**), and 7th day (**b**, **b′**) after the final ecdysis in both the normal and fasted aphid groups. In each of the group, a positive control sample were included to shown the cell apoptosis signals (**c**, **c′**). The arrowheads indicate apoptotic cells

In sections of aphid pterothorax stained in the TUNEL experiment, no apoptotic signals were observed in alatae aphid before the 7th day after eclosion (Fig. 2a). The first apoptotic signal appeared in aphids at the 7th day as brownish yellow grains (Fig. 2b), which is similar to the apoptosis signals in the positive controls (Fig. 2c, c′). In parallel, we did not find any apoptotic signals in the muscles of the 3rd, and 7th day fasted aphids (Fig. 2a′, b′).

#### Differentially expressed genes pre-/post-migration

We identified 36 differentially expressed genes that were classified into three groups (Additional file 6: Table S3): (1) genes related to apoptosis: this group includes the widely studied apoptotic genes like p53 regulator [15] and the apoptosis marker, *RPS27a* [7, 8]; Some additional genes are related to apoptosis in specific conditions. Ras-related protein Rab-29B regulates lysosome integrity related to age associated defects [16]. (2) genes related to metabolism: this group includes genes in metabolism of amino acids (e.g. d-amino acid dehydrogenase), sugars (e.g. glucose transporter 1), and vitamins (e.g. 3,4-dihydroxy-2-butanone-4-phosphate synthase). Genes important to energy re-allocation were also found differentially expressed, including exocyst complex component 3 [17] and serpin 3a [18]. (3) genes with unknown functions.

#### *Sitobion avenae RPS27a dynamic expression*

The *S. avenae RPS27a* encodes a 150 aa protein (76aa ubiquitin + 74aa ribosomal protein). The sequences of *RPS27a* are highly conserved across different insects (84–93.3%), and the divergence was mainly from the ribosomal protein, whereas the ubiquitin monomers were almost identical (Additional file 5: Figure S3).

**Fig. 3:**
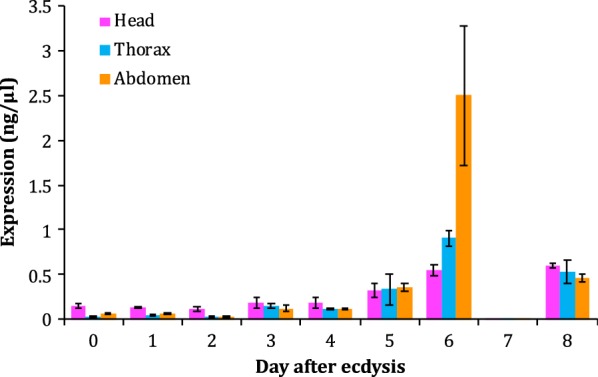
The dynamic expression of *S. avenae* ubiquitin-ribosomal S27a gene. The aphids we collected are from eclosion (0 day) to breeding finished (8th day). The expression in all three tissues always show stable and relatively lower, except a sharp increase in 5th day, with higher in abdomen than in head and thorax. Error bar = ± standard deviation

The expression of *S. avenae RPS27a* changed dynamically pre/post IFM degeneration. Initially after eclosion, *RPS27a* remained a constant low expression in head, thorax, and abdomen (Fig. 3). The expression started raising from the 3rd day post-eclosion. *RPS27a* expression significantly increased and was significantly higher in abdomen than head and thorax in the 6th day, which is 1 day post-migration and 1 day before the apoptotic signals were detected (Fig. 2c). After aphid migration, a sharp decrease of *RPS27a* occurred in the 7th day (Fig. 3), the day that apoptosis signals were detected (Fig. 2c).

### Discussion

IFM degeneration has been mostly studied physiologically in insects such as fruit fly, crickets, bugs, and some aphids [7, 19–25]. Here, we depicted the IFM development and degeneration in *S. avenae* and identified genes that may regulate IFM degeneration.

#### IFM degeneration for energy re-allocation

A trade-off has been proposed between IFM degeneration and reproduction with regard to energy allocation [26]. IFM degeneration is regulated in many insects, and the products are reallocated to reproduction [24–30]. Here, we identified two groups of genes that differentially expressed pre-/post- aphid migration (Additional file 6: Table S3). First group included apoptotic genes, like Ras-related Rab-29b, p53, and RPS27a, which suggested that aphid IFM degeneration is an active PCD. The second group included metabolic genes. Some of those genes are involved in the metabolism of energy building blocks (e.g. amino acids, sugars, and vitamins), while other genes are involved in energy transport (e.g. exocyst complex component-3). The differentially expressed genes indicated that aphid IFM degeneration is an active process that involves a trigger of PCD followed by a systematic reallocation of energies (e.g. from migration to reproduction) [5, 29, 31, 32].

#### Ubiquitin functions as a marker for aphid IFM degeneration

Ubiquitin degrades proteins in eukaryotic cells. During cell apoptosis, short-lived proteins are subjected to ubiquitination, which triggers different degenerative processes [33, 34]. Here, we found that the *S. avenae RPS27a* was significantly differentially expressed pre-/post aphid migration. *RPS27a* showed a sharp increase before aphid migration and a sudden decrease after migration, which indicated that RPS27a is programed during IFM degeneration. RPS27a may function as a trigger for apoptosis to regulate IFM degeneration [33–35] or RPS27a may degenerate waste proteins to facilitate energy reallocation [36]. Interestingly, from our qPCR results, *RPS27a* was primarily expressed in abdomen compared to head and thorax. During IFM degeneration, *RPS27a* may trigger a systematic energy reallocation during the shift from migration to reproduction; URS27a functions as a general protein degradation machinery throughout aphid body including abdomen, which is the major energy sink for reproduction.

## Limitations

The timing of migration and IFM degeneration determines whether insects can locate preferable host plants [37]. We investigated the IFM degeneration using tethered flight system on lab aphid population. However, the timing of aphid migration and IFM degeneration in nature remains elusive.

## Supplementary information

**Additional file 1: Figure S1.** Morphological and histological examinations of wing development in aphid nymphs. (A, a) The external and internal structures in the first instar nymphs under HD and LD conditions are similar. Wing primordia were observed in the internal structures of nymphs in both conditions. (B, b, b′) In the second instar stage, the external structures continued to be similar in nymphs under HD and LD conditions. However, the wing primordia (arrow) developed and enlarged in the winged line and the wing primordia disappeared in the wingless line. (C, C′, c, c′) Swollen structures were observed on the third instar nymphs under HD conditions, while not in the LD conditions. Swollen structures were identified as the wing bud in the future winged aphids. In the inner structures, the wing primordia continually developed and enlarged in the winged lines. Flight muscles fibers also differentiated. The primordia disappeared in the wingless line, and the area in which the flight muscle develops was occupied by fat bodies. (D, D′, d, d′) In the fourth instar stage, wing buds enlarged into the shape of a plate. There were no wing buds in the wingless line. The flight muscles of the winged line increased in size and occupied half of the thoracic area. The wing epithelia of the wing buds were folded in a complicated structure. The folding patterns were different between the forewings and hindwings. Fat bodies occupied the corresponding thoracic locations in the wingless aphids. (E) Wing hair sensilla were also seen in the winged line. (F) It shows the wing hair sensilla of the adult wing aphid.

**Additional file 2: Table S1.** DDRT-PCR primers.

**Additional file 3: Figure S2.** Gel visualization of differentially expressed genes before and after aphid migration. 6% polyacrylamide gel electrophoresis of PCR products from representative DDRT-PCR primer pairs. Arrowhead indicates genes that show different expressions before and after aphid migration.

**Additional file 4: Table S2.** Primers used for the amplification of RPS27a sequences.

**Additional file 5: Figure S3.** Alignment (left) and phylogenetic analysis (right) of amino acids sequences of ubiquitin-ribosomal S27a from18 insects. The tree shown is an observed divergency tree inferred from alignment. Statistical support for each individual node on the tree is shown above the nodes. GreenBox stands for Coleoptera, BlueBox stands for Lepidoptera, YellowBox stands for Diptera and RedBox stands for Hemiptera. *Al Agriotes lineatus (CAJ01876), Cg Carabus granulates (CAH04347), Md Micromalthus delibis (CAJ01880), Tc Tribolium castaneum (XP_969023), Tb Timarcha balearica (CAJ01881), Bm Byoxsm mori (ABM55591), Px Plutella xylostella (P68202), Pd Papilio dardanus (CAH04128), Sf Spodoptera frugiperda (P68203), Se Spodoptera exigua (Deduced), Dm Drosophila melanogaster (NP_476778), Aa Aedes aegypti(AAS79344), Dc Diaphorina citri (ABG81958) Ga Graphocephala atropunctata (DQ445503), Mh Maconellicoccus hirsutus(ABM55591), Of Oncopeltus fasciatus (ABN54483), AP Acyrthosiphon pisum (NP_001155610), Sa Sitobion avenae (Deduced)*.

**Additional file 6: Table S3.** Differentially expressed genes after aphid tethered migration.

## Data Availability

All data generated or analyzed during this study are included in this published article and its additional files.

## Abbreviations

RPS27a: ubiquitin-ribosomal S27a
IFM: indirect flight muscles
JH: juvenile hormone
TUNEL: Terminal deoxynucleotidyl transferase dUTP-biotin nick-end labeling assay
DDRT-PCR: differential-display reverse transcription-PCR
SEM: scanning electron microscope
PCD: programmed cell death
RACE: rapid amplification of cDNA ends
