# Supplementary figures and images for: Flight muscles degenerate by programmed cell death after migration in the wheat aphid, Sitobion avenae
Source: BMC Res Notes. 2019 Oct 21;12:672. doi: 10.1186/s13104-019-4708-z (PMC6805507; doi:10.1186/s13104-019-4708-z)

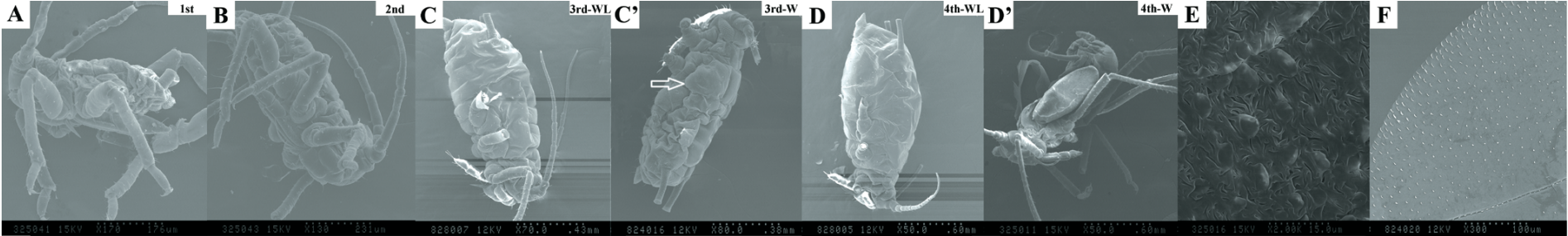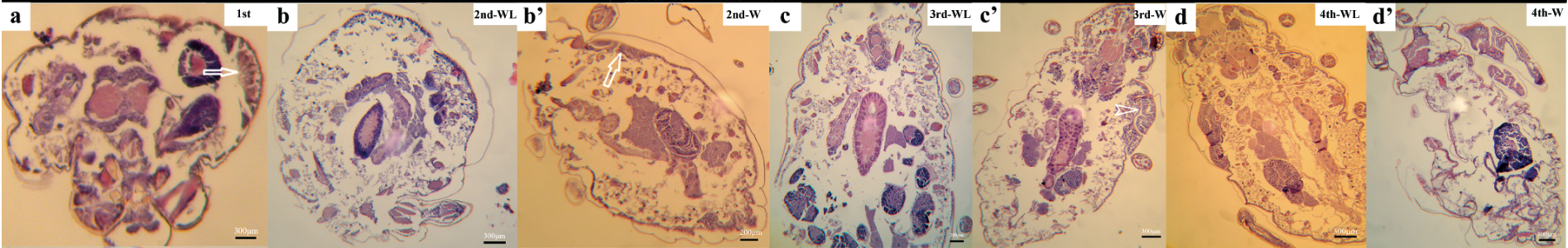

Supplement: Supplementary file 1 — Additional file 1: Figure S1. Morphological and histological examinations of wing development in aphid nymphs. (A, a) The external and internal structures in the first instar nymphs under HD and LD conditions are similar. Wing primordia were observed in the internal structures of nymphs in both conditions. (B, b, b′) In the second instar stage, the external structures continued to be similar in nymphs under HD and LD conditions. However, the wing primordia (arrow) developed and enlarged in the winged line and the wing primordia disappeared in the wingless line. (C, C′, c, c′) Swollen structures were observed on the third instar nymphs under HD conditions, while not in the LD conditions. Swollen structures were identified as the wing bud in the future winged aphids. In the inner structures, the wing primordia continually developed and enlarged in the winged lines. Flight muscles fibers also differentiated. The primordia disappeared in the wingless line, and the area in which the flight muscle develops was occupied by fat bodies. (D, D′, d, d′) In the fourth instar stage, wing buds enlarged into the shape of a plate. There were no wing buds in the wingless line. The flight muscles of the winged line increased in size and occupied half of the thoracic area. The wing epithelia of the wing buds were folded in a complicated structure. The folding patterns were different between the forewings and hindwings. Fat bodies occupied the corresponding thoracic locations in the wingless aphids. (E) Wing hair sensilla were also seen in the winged line. (F) It shows the wing hair sensilla of the adult wing aphid. [file 13104_2019_4708_MOESM1_ESM.pdf]

# 6% polyacrylamide gel

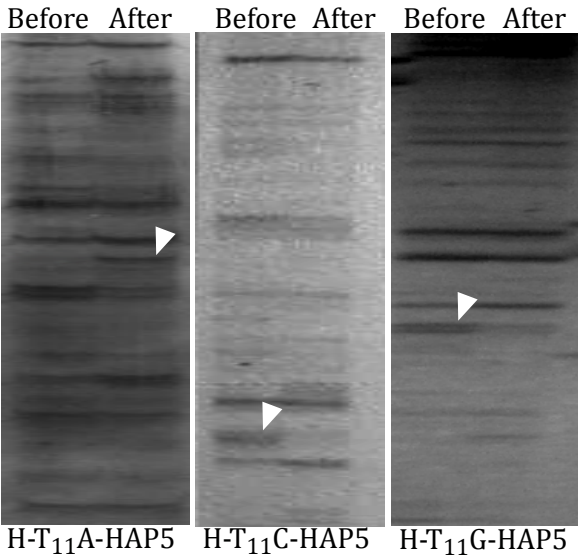

Supplement: Supplementary file 3 — Additional file 3: Figure S2. Gel visualization of differentially expressed genes before and after aphid migration. 6% polyacrylamide gel electrophoresis of PCR products from representative DDRT-PCR primer pairs. Arrowhead indicates genes that show different expressions before and after aphid migration. [file 13104_2019_4708_MOESM3_ESM.pdf]
