# Supplementary material for: Flight muscles degenerate by programmed cell death after migration in the wheat aphid, Sitobion avenae
Source: BMC Res Notes. 2019 Oct 21;12:672. doi: 10.1186/s13104-019-4708-z (PMC6805507; doi:10.1186/s13104-019-4708-z)
Supplement: Supplementary file 2 — Additional file 2: Table S1. DDRT-PCR primers. [file 13104_2019_4708_MOESM2_ESM.docx]

**Table S1** DDRT-PCR primers

| **3’- anchor primer** | **5’-random primer** |
| --- | --- |
| *AAGCT*TTTTTTTTTTTT*A*  *AAGCT*TTTTTTTTTTTT*C*  *AAGCT*TTTTTTTTTTTT*G* | *AAGCT*TGATTGCC  *AAGCT*TCGACTGT  *AAGCT*TTGGTCGA  *AAGCT*TCTCAACG  *AAGCT*TAGTAGGC  *AAGCT*TCGACCAT  *AAGCT*TAACGAGG  *AAGCT*TTTACCGC |
