# Supplementary material for: Flight muscles degenerate by programmed cell death after migration in the wheat aphid, Sitobion avenae
Source: BMC Res Notes. 2019 Oct 21;12:672. doi: 10.1186/s13104-019-4708-z (PMC6805507; doi:10.1186/s13104-019-4708-z)
Supplement: Supplementary file 4 — Additional file 4: Table S2. Primers used for the amplification of RPS27a sequences. [file 13104_2019_4708_MOESM4_ESM.docx]

**Table S2** Primers used for the amplification of Ub-RPS27a sequences

| **Primer name** | **Sequence (5’ to 3’)** | **Tm (°C)** |
| --- | --- | --- |
| Ub-F | ATGCARATYTTYGTIAARAC | 44.5 |
| Ub-R  GSP1  GSP2  Outer primer  Inner primer  qPCR-F  qPCR-R | CACTTBGTCCTYCGCCTGCG  GTGAAGACGTTGACTGGGAAAAC  CGAAGTAGAGTCGTCGGATTCAATT  TACCGTCGTTCCACTAGTGATTT  CGCGGATCCTCCACTAGTGATTTCACTATAGG  GTTGACTGGGAAAACGAT  CTAAGAACCAAGTGAAGG | 59.9  59.9  60.8  *  *  51.3  54.0 |

**Notes:** Ub-F: ubiquitin forward primer; Ub-R: ubiquitin reverse primer; GSP: gene special primer; qPCR-F: ubiquitin qPCR forward primer; qPCR-R: ubiquitin qPCR reverse primer; *: provided by Takara 3’-Full RACE Core kit; and all primers are synthesized by Sangon Biotech (Shanghai).
