# Supplementary material for: Flight muscles degenerate by programmed cell death after migration in the wheat aphid, Sitobion avenae
Source: BMC Res Notes. 2019 Oct 21;12:672. doi: 10.1186/s13104-019-4708-z (PMC6805507; doi:10.1186/s13104-019-4708-z)
Supplement: Supplementary file 5 — Additional file 5: Figure S3. Alignment (left) and phylogenetic analysis (right) of amino acids sequences of ubiquitin-ribosomal S27a from18 insects. The tree shown is an observed divergency tree inferred from alignment. Statistical support for each individual node on the tree is shown above the nodes. GreenBox stands for Coleoptera, BlueBox stands for Lepidoptera, YellowBox stands for Diptera and RedBox stands for Hemiptera. Al Agriotes lineatus (CAJ01876), Cg Carabus granulates (CAH04347), Md Micromalthus delibis (CAJ01880), Tc Tribolium castaneum (XP_969023), Tb Timarcha balearica (CAJ01881), Bm Byoxsm mori (ABM55591), Px Plutella xylostella (P68202), Pd Papilio dardanus (CAH04128), Sf Spodoptera frugiperda (P68203), Se Spodoptera exigua (Deduced), Dm Drosophila melanogaster (NP_476778), Aa Aedes aegypti(AAS79344), Dc Diaphorina citri (ABG81958) Ga Graphocephala atropunctata (DQ445503), Mh Maconellicoccus hirsutus(ABM55591), Of Oncopeltus fasciatus (ABN54483), AP Acyrthosiphon pisum (NP_001155610), Sa Sitobion avenae (Deduced). [file 13104_2019_4708_MOESM5_ESM.pdf]

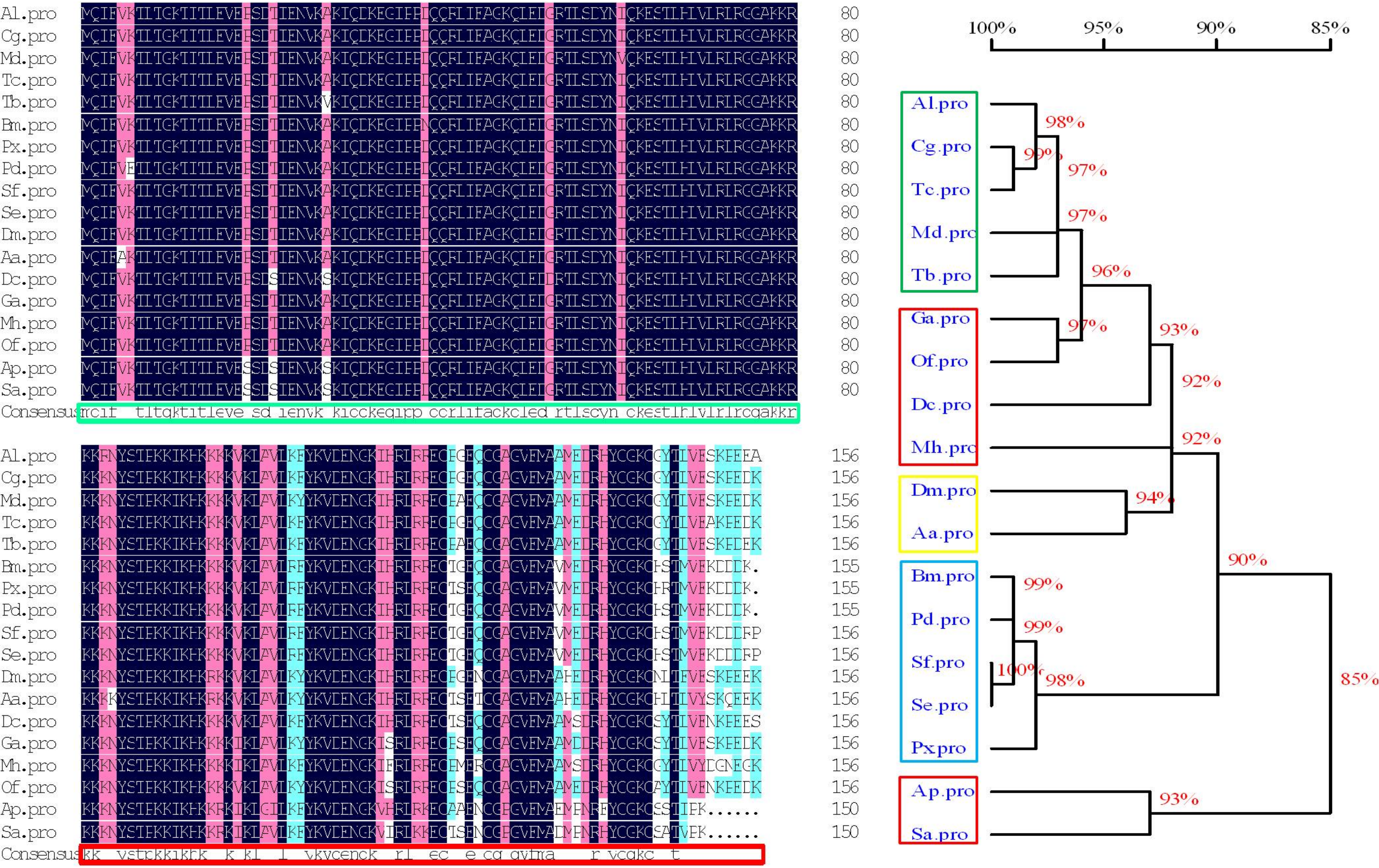

Al.pro
Cg.pro
Md.pro
Tc.pro
Tb.pro
Bm.pro
Px.pro
Pd.pro
Sf.pro
Se.pro
Dm.pro
Aa.pro
Dc.pro
Ga.pro
Mh.pro
Of.pro
Ap.pro
Sa.pro
Consensus
80
156
155
150
100%
95%
90%
85%
98%
99%
97%
96%
93%
92%
94%
90%
100%
85%
