# Supplementary material for: Flight muscles degenerate by programmed cell death after migration in the wheat aphid, Sitobion avenae
Source: BMC Res Notes. 2019 Oct 21;12:672. doi: 10.1186/s13104-019-4708-z (PMC6805507; doi:10.1186/s13104-019-4708-z)
Supplement: Supplementary file 6 — Additional file 6: Table S3. Differentially expressed genes after aphid tethered migration. [file 13104_2019_4708_MOESM6_ESM.docx]

**Table S3** Differentially expressed genes after aphid tethered migration

| **NCBI Top Hit** | **Hit Species** | **Annotation** |
| --- | --- | --- |
| **Genes related to Apoptosis:** |  |  |
| Ras-related protein Rab-39B-like | *A. pisum* | Autophagy, Signal Transduction, intracellular transport |
| P53-like regulator | *D. melanogaster* | DNA repair, Apoptosis and cell cycle |
| Cisplatin resistance-associated overexpressed protein | *A. pisum* | Resistant to apoptosis that caused by accumulating cisplatin |
| Chaperone Hsp10 | *Hamiltonella defensa* | Relate to apoptosis |
| Riboflavin binding hexamerin precursor | *Hyalophora cecropia* | pupal-adult morphogenesis |
| LIM/homeobox protein Lhx5-like | *A. pisum* | Morphogenesis |
| Ubiquitin ribosomal S27a-like | *A. pisum* | Protein degradation |
| **Genes related to metabolism:** |  |  |
| D-amino-acid dehydrogenase | *Variovorax paradoxus* | Oxidation of D-amino acids to oxoacids |
| Beta-galactosidase, subunit alpha | *Escherichia albertii* | Hydrolyze galactose and its organic moiety |
| Glucose transporter 1 | *Macrosiphum avenae* | Glucose transport |
| Probable maltase L-like | *A. pisum* | Maltose hydrolysis |
| Trifunctional nucleotide phosphoesterase YfkN | *A. pisum* | Catalyze the release of inorganic phosphate from 2’,3’-cyclic nucleotides |
| UDP-N-acetylmuramate dehydrogenase | *Megamonas hypermegale* | Aminosugars metabolism |
| 3,4-dihydroxy-2-butanone-4-phosphate synthase | *Haemophilus influenzae* | Riboflavin synthesis |
| RelA/SpoT domain protein | *Treponema vincentii* | synthesize and/or hydrolyze alarmone ppGpp |
| zinc finger protein 706-like | *A. pisum* | Regulation of translation |
| Eukaryotic translation initiation factor 5-like | *A. pisum* | Initiation of eukaryotic translation |
| Staphylococcal nuclease domain-containing protein 1 | *A. pisum* | Mediate miRNA decay |
| Serpin 3a | *Nasonia vitripennis* | Protease inhibition |
| Putative 115 kDa protein in type I Retrotransposable element R1DM | *Nasonia vitripennis* | Retrotransposon |
| SET and MYND domain-containing protein 4-like | *A. pisum* | Metal ion binding and methyltransferase activity |
| AP-2 complex subunit mu-1-like | *A. pisum* | Endocytosis, cargo selection and vesicle formation |
| Exocyst complex component 3-like | *A. pisum* | Regulated exocytosis |
| Clavesin-1-like | *A. pisum* | Lysosome and endosomes organization |
| Lanthionine synthetase C-like | *Clostridium cellulovorans* | Peptide modifying enzyme |
| Aerobactin biosynthesis protein, LucA/LucC | *Staphylococcus hominis* | Siderophore biosynthesis |
| P450-4 for ent-kaurene oxidase | *Gibberella moniliformis* | Gibberellins biosynthesis |
| Aphid transmission protein | *Barley yellow dwarf virus* | Virus Transmission |
| **Genes with unknown function:** |  |  |
| Conserved Plasmodium protein | *Plasmodium falciparum* | Unknown |
| BAC VMRC38-20-A10 | *A. pisum* | Unknown |
| hypothetical protein LOC100570547 | *A. pisum* | Unknown |
| Chromosome 16 clone CTD-2318B16 | *Homo sapiens* | Unknown |
| ACI0AAF20YG23, full-insert cDNA sequence | *A. pisum* | Unknown |
| ACI0AAF4YA05, full-insert cDNA sequence | *A. pisum* | Unknown |
